# Obtaining retrotransposon sequences, analysis of their genomic distribution and use of retrotransposon-derived genetic markers in lentil (*Lens culinaris* Medik.)

**DOI:** 10.1371/journal.pone.0176728

**Published:** 2017-04-27

**Authors:** Rita Rey-Baños, Luis E. Sáenz de Miera, Pedro García, Marcelino Pérez de la Vega

**Affiliations:** Área de Genética, Dpto. de Biología Molecular, Universidad de León, León, Spain; University of Helsinki, FINLAND

## Abstract

Retrotransposons with long terminal repeats (LTR-RTs) are widespread mobile elements in eukaryotic genomes. We obtained a total of 81 partial LTR-RT sequences from lentil corresponding to internal retrotransposon components and LTRs. Sequences were obtained by PCR from genomic DNA. Approximately 37% of the LTR-RT internal sequences presented premature stop codons, pointing out that these elements must be non-autonomous. LTR sequences were obtained using the iPBS technique which amplifies sequences between LTR-RTs. A total of 193 retrotransposon-derived genetic markers, mainly iPBS, were used to obtain a genetic linkage map from 94 F_7_ inbred recombinant lines derived from the cross between the cultivar Lupa and the wild ancestor *L*. *culinaris* subsp. *orientalis*. The genetic map included 136 markers located in eight linkage groups. Clusters of tightly linked retrotransposon-derived markers were detected in linkage groups LG1, LG2, and LG6, hence denoting a non-random genomic distribution. Phylogenetic analyses identified the LTR-RT families in which internal and LTR sequences are included. *Ty3-gypsy* elements were more frequent than *Ty1-copia*, mainly due to the high *Ogre* element frequency in lentil, as also occurs in other species of the tribe Vicieae. LTR and internal sequences were used to analyze *in silico* their distribution among the contigs of the lentil draft genome. Up to 8.8% of the lentil contigs evidenced the presence of at least one LTR-RT similar sequence. A statistical analysis suggested a non-random distribution of these elements within of the lentil genome. In most cases (between 97% and 72%, depending on the LTR-RT type) none of the internal sequences flanked by the LTR sequence pair was detected, suggesting that defective and non-autonomous LTR-RTs are very frequent in lentil. Results support that LTR-RTs are abundant and widespread throughout of the lentil genome and that they are a suitable source of genetic markers useful to carry out further genetic analyses.

## Introduction

Lentil (*Lens culinaris* Medik. subsp. *culinaris*) is one of the earliest domesticated plant species in the Fertile Crescent. It is a diploid (2n = 14), self-pollinated annual cool season grain legume normally grown in temperate semi-arid regions, usually in rotation with cereals. It plays an important role in human nutrition and soil improvement contributing to replenish the soil nitrogen levels. The crop is now widely cultivated throughout Western Asia, Northern Africa, the Indian subcontinent, Australia and North America [1–2]. This species is included in the tribe Fabeae or Vicieae, which also includes the genera *Lathyrus*, *Pisum*, *Vavilovia* and *Vicia* [3–4].

**Fig 1:**
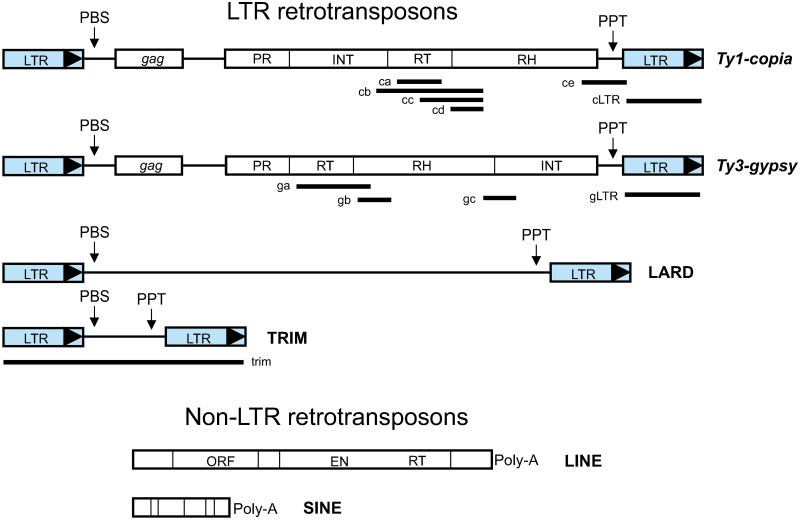
Schematic representation of representative LTR retrotransposons. The main characteristics of autonomous and non-autonomous elements are represented. LTR-retrotransposons have long terminal repeats (LTRs) in direct orientation. Autonomous elements contain at least two genes, called *gag* and *pol*. The *gag* gene encodes a capsid-like protein and the *pol* gene encodes a polyprotein that is responsible for protease (PR), reverse transcriptase (RT), RNase H (RH) and integrase (INT) activities. PBS, primer binding site; PPT, polypurine track. Non-autonomous elements, such as large retrotransposon derivatives (LARDs) and terminal repeat retrotransposons in miniature (TRIMs), lack most or all coding sequence. Non-LTR retrotransposons are divided into long interspersed nuclear elements (LINEs) and short interspersed nuclear elements (SINEs). LINE coding regions include a gag-like protein (ORF), an endonuclease (EN) and reverse transcriptase (RT). Both LINEs and SINEs usually terminate by a poly(A) sequence [5]. Thick lines below the elements indicate the sequences amplified in lentil in this work; the first letter c in the nomenclature indicates that the sequence was identified as a *copia* and g as a *gypsy* element. Drawings not made to scale.

Transposable elements (TEs) are DNA sequences that can insert into new chromosomal locations and often make duplicate copies of themselves in the process. TEs are the single largest component of most eukaryotic genomes; although active elements comprise only a small minority of the genomic TE complement in most multicellular organisms. Eukaryotic TEs are divided into two classes according to whether their transposition intermediate is RNA (class 1, or retrotransposons) or DNA (class 2). Class 1 elements are classified into two groups depending upon the presence or not of long terminal repeats (LTRs): LTR retrotransposons and non-LTR retrotransposons [5–6] (see Fig 1). Retrotransposons (RTs) are the most abundant and widespread class of eukaryotic TEs and are widely distributed along plant genomes [6–7]. The plant genome percentage represented by RTs in assembled genomes was found to range between 7.0 of *Populus trichocarpa* to 75.6 of *Zea mays*. In the legume model species *Medicago truncatula* RT coverage corresponded to 26% and in the chickpea crop (*Cicer arietinum*) amounted to 49% [8], while in the Vicieae species comprised up to 81% of the nuclear genome [9].

LTR retrotransposons (LTR-RTs) are further classified into the *Ty1-copia* and *Ty3-gypsy* families that differ from each other in both their sequences similarity degree and encoded gene product order [6], and into two groups almost exclusive to plants, LARDs (LArge Retrotransposon Derivatives) and TRIMs (Terminal-repeat Retrotranposons In Miniature) [10–11]. LARDs are approximately 4.4 kb long but they do no but contain no open reading frames encoding typical retrotransposon proteins [10]; while TRIMs have terminal direct repeat sequences that encompass an internal domain of 100–300 bp [11] (Fig 1).

Among genomes of the species belonging to the tribe Vicieae LTR-RT elements are predominant, reaching approximately 140,000 copies per genome in pea (*Pisum sativum*); *Ty3-gypsy* elements are less diverse and have accumulated to a higher copy number compared to *Ty1-copia* elements. This is in part due to a large proportion of *Ogre*-like retrotransposons, included into the *Ty3-gysy* elements, which alone can make up more than 50% of the genome in some species of this tribe. *Ogre* elements are exceptionally large sized (reaching up to 25 kb) and possess several specific features. The *Ty1-copia* group elements are somewhat less abundant with only *Maximus/SIRE* elements reaching the abundance of some of the *Ty3-gypsy* lineages, and LARD and TRIM elements are present in even lower amounts [12–14].

The relative high copy number of retrotransposons coupled to their high genome mobility has consequently generated a relatively high number of polymorphisms. Thus, retrotransposon sequences have been used to develop several types of genetic markers such as sequence specific amplified polymorphisms (SSAP), also known as transposon display, inter-retrotransposon amplified polymorphisms (IRAP), retrotransposon-microsatellite amplified polymorphisms (REMAP), retrotransposon based insertion polymorphisms (RBIP), inter-primer binding site (iPBS) and others [15–18]. These markers have been used for phylogenetic studies and have been included in numerous genetic maps of several crop plant species [19], having proved their utility in the molecular dissection of plant genomes, genetics and breeding [20]. In the Vicieae species they have been used in *Lens* diversity and phylogenetic studies [21] as well as in other genera [22–28].

The aims of this work were to identify some of the LTR-RT elements present in lentil (*Lens culinaris* Medik.) by partial element sequencing and to examine their distribution throughout of the lentil draft genome [29], as well as to generate retrotransposon derived markers in this species for their inclusion in genetic maps. These resultant markers can be useful in future genetic studies and in lentil breeding by marker-assisted selection, in addition to LTR-RT sequences aiding in the assembly of the lentil draft genome.

## Material and methods

### Plant material

The material used for the genetic mapping was a set of 94 F_7_ inbred recombinant lines (RILs) derived from the cross between the cultivar Lupa and the wild lentil ancestor *L*. *culinaris* subsp. *orientalis* (Bioiss.) Ponert (Spanish Germplasm Bank accession BG16880). The DNA sequences of the LTR and the internal RT components were derived from the cultivar Lupa.

### Marker analysis

DNA was extracted from leaves of two-three week old seedlings with the Dnaesy Plant Mini Kit (Quiagen) following the manufacturer’s instructions. DNA quantification was carried out with a NanoDrop ND-1000 (Thermo Fisher). Putative retrotransposon sequences were amplified from genomic DNA by PCR, the results analyzed by means of 1–2% agarose gels and the selected bands were isolated, cloned and sequenced using the Sanger method and capillary electrophoresis (MegaBACE 500 Amersahm Biosiences). The PCR, electrophoresis in agarose gels, cloning and sequencing techniques used comprised standard methods [30]. Reverse transcriptase sequences were amplified using the degenerated primers designed for the *Ty1-copia* [31], for the *Ty3*-*gypsy* [32], or designed from a lentil *Ty1-copia* retrotransposon Tnana [33] (S1 Table).

The iPBS markers were amplified using 12 primers previously described [17] (S1 Table). The inter-primer binding site technique uses a single primer (sometimes two) to amplify the sequences enclosed between the PBS sites of two nearby LTR-RTs displaying inverted orientations. Additional markers were obtained with a primer described by Hamwieh et al. [34] and two microsatellite primers, (AC)_10_ and (GT)_10_, in combination with primers derived from lentil retrotransposons (S1 Table). Three additional partial sequences corresponding to the RNase H were obtained using previously described primers [35]. Lentil genomic DNA was digested with *Mse*I, next *Mse*I adapters were added and linked to the corresponding primers used for the PCR reactions (S1 Table). The GeneBank lentil sequence accession numbers correspond to KX871706 for Tnana, and KX889312 to KX889392 in the case of the rest.

### Genetic mapping

Genetic maps were obtained with the software packages MapMaker v 3.0b [36], CarthaGene 1.3.beta [37] and MapChart v 2.2 [38]. Evolutionary analyses were conducted in MEGA7 [39]. Dendrograms were obtained using the neighbor-joining algorithm [40], making use of the Tamura 3 parameter distance [41] and the gamma distribution in order to consider the substitution rate differences among sites.

### Genomic analysis

Some of the retrotransposon lentil sequences, internal or from the LTRs, were used to carry out an *in silico* search of homologous sequences appearing in the current lentil draft genome v0.8 [29]. The search was carried out using the BLASTn v 2.3.0 [42], the “outformat” option used entailed the single line per query one. Since the sequences used as *in silico* “probes” were derived from different retroelement parts, two or more of them could identify by BLAST the same genomic element. In order to avoid different “probes” to return repeated hits of the same element the following procedure was carried out. 1) Within of each contig all of the sequences identified were aligned on a single strand, 2) Within of each contig the sequences were ordered considering the “start” and the “end” sites, 3) Two or more hits were considered as part of the same retroelement if they overlapped or if the distance between the consecutive “end” and “start” sites was not longer than a determined distance; for the non-overlapping sequences four distance categories were considered: 10, 1,000, 10,000 or 50,000 bp. Statistical analyses were performed with the R software [43].

## Results

### Genetic markers and mapping

The iPBS markers were used in the genetic linkage analysis and to sequence and identify some retrotransposon LTRs. Primers complementary to the retrotransposon primer binding site (PBS) located close to the 5’ LTR sequence, were used to amplify the LTRs and the spacer region between retrotransposons (RT). All primers and primer-pair combinations were tested (PBS1 to PBS12, S1 Table), all of them except PBS11 generated polymorphic markers between the two parents. In total 741 bands were scored of which 233 iPBS evidenced polymorphism between *L*. *c*. *culinaris* and *L*. *c*. *orientalis* (amplicon sizes ranged from 400 to 2,000 bp). Additional genetic markers were obtained using primers designed from the lentil sequences obtained in this work, belonging to *Angela* (LTR1) and to *SIRE1/Maximus* (LTR3) of the *Ty1-copia* family, and to *Peabody* (LTR4) and to TRIM type *Cassandra* (LTR2) of the *Ty3-gypsy* type family. Likewise, markers with flanking lentil microsatellites (SSR), together with primers for SSR sequences (S1 Table) were obtained. Clear polymorphic bands were only observed for the primer combinations LTR1-AC, LTR4-GT and LTR2-SSR66R.

**Fig 2:**
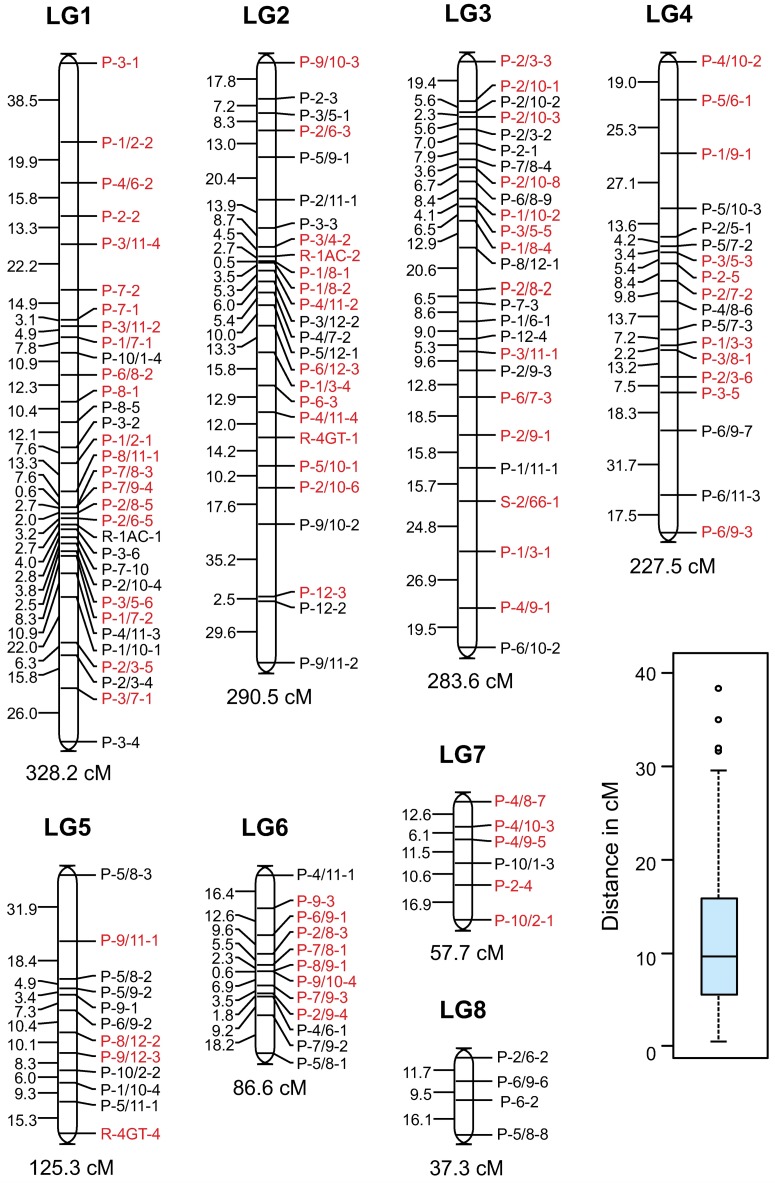
Genetic map obtained with markers showing Mendelian segregations. The markers from the parental *L*. *culinaris* Lupa are indicated in red, that is, these bands were observed in the parental Lupa but were absent in the other parental, and vice versa for markers in black. Linkage groups are numbered from LG1 to LG8. A LOD score of 4 was used. Markers preceded by a P are iPBSs, by R are REMAPs and S indicates the SSR markers included. Partial distances in cM are indicated to the left of LGs while the total LG distance is displayed at the bottom. The insert to the right corresponds to the boxplot distribution of the distance in cM between consecutive markers.

While the iPBS technique yielded a relatively high number of markers (a total 233 polymorphic markers, representing approximately 3.5 markers per primer combination), the number of markers obtained from the other primer combinations was low. One hundred and eighty four iPBS, six REMAP and three markers derived from the SSR66R primer showed a 1:1 segregation in the F_7_ RIL population analyzed. A total of 131 iPBS, four REMAP and one SSR66 marker were placed in a recombination genetic map encompassing eight linkage groups (Fig 2). Three linkage groups holding only two markers were not considered to carry out the analysis. The linkage groups ranged from 328.2 cM to 37.3 cM, including 32 to four markers, respectively. Distances between the consecutive markers varied from 0.5 to 38.5 cM, with an average of 11.2 cM. As can be seen in the box-plot included in Fig 2, most distances were lower than 15 cM and included in the third first quartiles (median of 9.6 cM) and distances higher than 30 cM could be considered as outliers. In case of not taking into account markers positioned at distances near to 30 cM or higher, some markers located near to the end of linkage groups ends would have been considered as independent. Clusters of tightly linked retrotransposon derived markers were detected in groups LG1, LG2, and LG6 (Fig 2).

### LTRs and internal retrotransposon sequences

Internal parts of the retrotransposons were also amplified using degenerate and non-degenerate primers (S1 Table). Using the primers described for *Ty1-copia* elements [31], two PCR bands were cloned and sequenced. The smaller band of ~ 280 bp yielded 21 different sequences derived from 23 clones (Table 1 and S1 Fig). The second band of ~ 450 bp yielded six different sequences out of 12 clones, which after sequencing were identified as *Ty3-gypsy* elements. All the sequences corresponded to reverse transcriptase. The primers for *Ty3*-*gypsy* elements [32] yielded five discrete bands, ranging from ~ 850 bp to 2500 bp. From 55 clones, 22 different sequences were identified, corresponding to reverse transcriptase and some of them also overlapped with the beginning of the downstream RNase H. Further analyses included the *Ty3*-*gypsy* sequences obtained from the *Ty1-copia* primers in the *Ty3*-*gypsy* data set (Table 1 and S2 Fig). The position of the cloned DNA fragments in the RTs are indicated in Fig 1.

**Table 1:** Partial retrotransposon sequences obtained from lentil cultivar Lupa.

Primers	Amplified family	Clone nomenclature (length in bp)^1^
Copia (ca)^2^	*Copia* Gr1^3^	Copia-304^4^, Copia-306, Copia-307, Copia-311, Copia-316, Copia-321, (276)
	*Copia* Gr2	Copia-301, Copia-320, Copia-315, Copia-318 (279)
	*Copia* Gr3	Copia-314 (259), Copia-317 (276), Copia-322 (274)
	*Copia* Gr4	Copia-302, Copia-303 (279)
	*Copia* Gr5	Copia-323 (276)
	*Copia* Gr6	Copia-305, Copia-308, Copia-310, Copia-312 (276)
	*Copia* Gr7	Copia-319 (273)
Gypsy (ga)	*Gypsy* Gr3a	Gypsy3-408 (417)
	*Gypsy* Gr3b	Gypsy3-305 (417)
Gypsy (ga)	*Gypsy* Gr1a	Gypsy1-104, Gypsy1-304 (816), Gypsy1-307 (252), Gypsy1-314 (251), Gypsy1-402 (264)
	*Gypsy* Gr1b	Gypsy1-306 (418), Gypsy-401 (417)
	*Gypsy* Gr1c	Gypsy1-105, Gypsy1-313 (543)
	*Gypsy* Gr1d	Gypsy1-412 (371), Gypsy1-308 (542), Gypsy1-405 (852), Gypsy1-415 (877)
	*Gypsy* Gr1e	Gypsy1-407 (623)
	*Gypsy* Gr2	Gypsy2-409 (889)
	*Gypsy* Gr3b	Gypsy3-201 (381), Gypsy3-203, Gypsy3-512 (207), Gypsy3-501 (276), Gypsy3-416 (415)
Copia (ga)	*Gypsy* Gr1c	Gypsy1-212 (258)
	*Gypsy* Gr1e	Gypsy1-201, Gypsy1-203, Gypsy1-208 (442), Gypsy1-207 (441), Gypsy1-210 (386)
Tnana (cb)	*Copia*	TnanaC-112 (1455)
Tnana (cc)	*Copia*	TnanaC-241, TnanaC-242, TnanaC-244, TnanaC-245 (719)
Tnana (cd)	*Copia*	TnanaC-343, TnanaC-345, TnanaC-352, TnanaC-356, TnanaC-357, TnanaC-359 (642), TnanaC-224 (340)
Tnana (cc)	*Copia*	TnanaC-311 (675), TnanaC-312 (688), TnanaC-334(628)
Tnana (gb)	*Gypsy*	TnanaG-121, TnanaG-123, TnanaG-125, TnanaG-353 (728), TnanaG-341, TnanaG-358 (727), TnanaG-235 (730), TnanaG-367(544)
RNaseH/MseI (ce)	Copia	RNaseC-13 (537), RNaseC-24 (321)
RNaseH/MseI (gc)	*Gypsy*	RNaseG-22 (666)

^1^ Lengths in bp (within parentheses) refer to all afore written sequences.

^2^ Letters between parentheses indicate the retrotransposon region amplified as represented in Fig 2. See supplementary Table 1 for primers used.

^3^ Gr nomenclature refers to the dendrogram groups of Fig 3.

^4^ Sequences underlined were used in the *in silico* search of the lentil draft genome.

**Fig 3:**
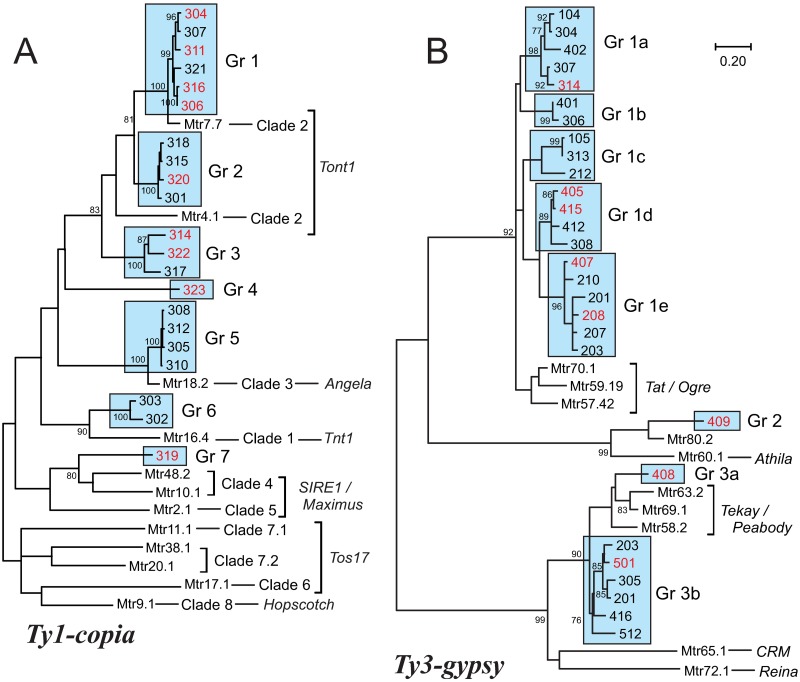
Phylogenetic trees of reverse transcriptase sequences. Trees show the relationships between lentil sequences and *Medicago truncatula* (Mtr) sequences. A, *Ty1-copia* sequences; B, *Ty3-gypsy* sequences. Lentil sequences are within boxes indicating the different linkage groups (Gr) to which they belong, groups were related to the *M*. *truncatula* clades as described by Piednöel et al. [46] and the *M*. *truncatula* sequence numbers as in Wang and Liu [44]. Red color denotes the presence of premature stop codons in the reading frames.

All sequences were aligned and then compared with *Medicago truncatula* LTR retrotransposons [44], those with a high similarity (e value < 10^−20^) were included in the subsequent analysis. Twenty-one sequences belonged to the *copia* family and all of them shared the conserved amino acid motif SLYGLKQA characteristic of the *copia* elements [31, 45] (S1 Fig). A neighbor-joining dendrogram was constructed in which the *copia* sequences were distributed among six sequence groups (Fig 3A) whose phylogenetic lineages were identified following the classification by Piednöel et al. [46] as *Tnt1/Angela/Tont1*, and *SIRE1/Maximus*. The 28 *gypsy* sequences were clustered in three groups (Fig 3B) classified as *Tat/Ogre*, *Athila* and *Tekay/DEL/Peabody*. Sequences of Group 3 shared several conserved amino acid motifs: LRID, DLRSGY, FG, and FIDD; while sequences in Group 1 shared variants of these sequence patterns: PHID, DGFSGY, FG, and YVDD (S2 Fig). The sequences considered in this analysis amplified from the *gypsy* and *copia* retrotransposons are listed in Table 1 and their corresponding positions within retroelements are indicated in Fig 1.

The three primer combinations (the reverse primer was common to all) derived from the lentil *copia* element named Tnana amplified between two to five bands per combination allowing to sequence a total of 23 different PCR products (Table 1). After the alignment and phylogenetic analyses, these sequences were identified as part of the two major groups of plant retrotransposons. Fifteen come from of the *pol* gene of *Ty1-copia* sequences, similar to the *Mtr*38.1 sequence of *M*. *truncatula* included in the *Tos17* group (Fig 3A), and eight were partial sequences of the RNase H *Ty3-gypsy*, similar to the *Mtr*59.19 included in the *Tat* group (Fig 3B). In addition to the presence of premature stop codons all these sequences evidenced changes in the described conserved active sites of the different enzymatic activities of the polyprotein, thus indicating that they must correspond to non-autonomous elements.

Two of the three partial sequences of the RNase H, encompassing from the end of the RNase H to the LTR (segment ce in Fig 1), were included in the *copia* family while the third (segment gc in Fig 1) was included in the *gypsy* family.

A total of 28 sequences out of the 75 retrieved that corresponded to the internal segment included premature stop codons in their ORFs. These codons were generated by point mutations or, more frequently, by frameshift mutations due to indels, generally deletions of several tens of nucleotides. These clones are marked with an asterisk in Fig 3.

Several iPBS markers were partially sequenced and those that had a high similarity with different retrotransposon families were further sequenced in order to obtain their complete sequences. Finally, six sequence types were selected to undertake a further analysis of their distribution throughout of the *Lens* genome. A complete LTR with similarity to an *Angela* lentil sequence [47] was amplified using the PBS7 primer; three LTRs of the non-autonomous TRIM *Cassandra* were amplified using PBS1, PBS2 or PBS2-PBS3; two complete LTRs of the *Ty1-copia* family *SIRE1/Maximus* similar to the gmw2-109b11-re-3 element of *Glycine max* [48] were obtained from primers PBS1-PBS3 and PBS3-PBS5; three LTRs of a *SIRE1-13* element (*Ty1*-*copia*) [49] were retrieved from PBS1, PBS7 and PBS2-PBS3; three sequences similar to the LTR of *Peabody* elements (family *Ty3-gypsy*) were recovered from PBS1, PBS3 and PBS1-PBS2; and finally, part of the LTR of an *Ogre* element (family *Ty3*-gypsy) was derived using PBS1-PBS7 (Table 2 and localization in Fig 1).

**Table 2:** Retrotransposon long terminal repeat sequences amplified from lentil cultivar Lupa.

LTR name (*Lineage*)	Family	Primers (length in bp)^1^
LTR-Angela (*Angela*)	*Copia* (cLTR)^2^	PBS 7 (1273)
LTR-Angela-S^3^ (*Angela*)	*Copia* (cLTR)	LTR-Ps/LTR-1F (1492)
LTR-Glycine (*SIRE1/Maximus*)	*Copia* (cLTR)	PBS 1/3 (267), PBS 3/5 (253)
LTR-SIRE (*SIRE1/Maximus*)	*Copia* (cLTR)	PBS 2/3 (826), PBS 3 (792), PBS 7 (644)
LTR-Peabody (*Tekay/DEL/Peabody*)	*Gypsy* (gLTR)	PBS 1 (1358), PBS 3 (861), PBS 1/2 (804)
LTR-Ogre (*Tat/Ogre*)	*Gypsy* (gLTR)	PBS 1/7 (172)
LTR-Cassandra (*TRIM*)	*TRIM* (trim)	PBS 1 (417), PBS 2 (415), PBS 2/3 (391)

^1^ Length in bp (within parentheses) refers to all afore written sequences.

^2^ Letters between parentheses indicate the retrotransposon region amplified as represented in Fig 2.

^3^ Sequence derived from Smýkal et al. (2009).

Likewise, the lentil LTR sequence described by Smykal et al. [47] was amplified using the primers described by these authors. We obtained a similar *Angela* LTR sequence, although it differed mainly in short insertion-deletions (Table 2).

### Genomic distribution

The sequences listed in Tables 1 and 2 were used as “probes” in an *in silico* BLASTn search against the lentil draft genome v 0.8 [29]. This draft of the *L*. *culinaris* genome includes 490,452 contigs with an average length of 5,673 bp, ranging between 64 bp to 605,900 bp. Only sequences with an e-value lower than 3*10^−4^ were considered. The results obtained with the different “probe” sequences or groups of them are summarized in Table 3. Depending upon the distance between two consecutive hits they were either considered as part of the same element or included in two different elements, the number of putative retrotransposons identified ranged from 31,216 to 24,633 (Table 3) when the internal parts of retrotransposons were used as “probes”. When the LTRs were used, the range was between 51,027 to 30,968. Thus, the LTRs identified a higher number of elements in spite of existing two per element and the number of LTR used as a probe being lower than the internal sequence probes. Likewise, the LTR data revealed that the *SIRE* lineage was the most abundant class among the *Ty1*-*copia* elements, representing 74% to 81% of the *copia* elements depending on the distance considered between hits. *Ogre* elements were the most abundant among the *Ty3*-*gypsy*, although in a lower percentage (57% to 65% of the *gypsy* sequences), followed by *Peabody* elements. *Ogre* retrotransposons constitute large elements [14] so that the number of the different *Ogre* elements identified in the lentil contigs could be close to the figure displayed in the fourth column of Table 3 considering 50,000 bp distances between consecutive hits.

**Table 3:** Number of *in silico* hits of lentil retrotransposon sequence types in relation to distances between consecutive hits.

“Probes”	Distance (in bp)				Number of Contigs
	> 10	> 1,000	> 10,000	> 50,000	
*Ty1-copia* Rtr^1^ Group 1	27	27	26	26	26
*Ty1-copia* Rtr Group 2	82	82	81	81	81
*Ty1-copia* Rtr Group 3	88	88	86	85	85
*Ty1-copia* Rtr Group 4	35	35	34	34	34
*Ty1-copia* Rtr Group 5	14	14	14	13	13
*Ty1-copia* Rtr Group 6	349	347	341	337	337
*Ty1-copia* Rtr Group 7	18	18	18	18	18
*Ty1-copia* Rtr	626	624	612	605	605^2^
*Ty3-gypsy* Rtr Group 1a	4,772	4,566	4,485	4,362	4,326
*Ty3-gypsy* Rtr Group 1b	789	783	771	761	761
*Ty3-gypsy* Rtr Group 1c	2,292	2,264	2,233	2,190	2,175
*Ty3-gypsy* Rtr Group 1d	4,489	4,363	4,271	4,131	4,079
*Ty3-gypsy* Rtr Group 1e	5,207	4,918	4,847	4,736	4,701
*Ty3-gypsy* Rtr Group 2	27	27	25	25	25
*Ty3-gypsy* Rtr Group 3a	3,079	3,036	2,967	2,907	2,892
*Ty3-gypsy* Rtr Group 3b	7,968	7,875	7,482	7,101	6,974
*Ty3-gypsy* Rtr	24,656	23,857	22,727	20,384	19,715^2^
Tnana copia	274	263	168	163	163
Tnana gypsy	15,064	14,507	14,078	13,757	13,677
RNaseH copia	478	478	472	463	463
RNaseH gypsy	3,515	3,456	3,410	3,356	3,341
*Ty1-copia*	1,378	1,365	1,251	1,224	1,220
*Ty3-gypy*	29,838	27,937	26,442	23,706	22,971
**All internal probes**	**31,216**	**29,301**	**27,624**	**24,633**	**23,810**^2^
LTR-Angela	3,709	2,521	2,239	2,106	2,079
LTR-Smykal	2,448	2,370	2,086	1,965	1,941
LTR-Glycine	26	26	20	20	20
LTR-SIRE	10,454	10,331	8,696	7,847	7,659
*Ty1-copia* LTR	14,144	13,090	10,898 (25.7%)	9,680	9,409^2^
LTR-Peabody	15,529	14,964	12,885	11,756	11,467
LTR-Ogre	20,802	20,618	19,135	16,475	15,913
*Ty3-gypsy* LTR	36,323	35,451	30,559 (72.0%)	25,193	24,096^2^
LTR-Cassandra (*TRIM*)	1,141	1,062	998 (2.3%)	979	973
**All LTR probes**	**51,027**	**49,351**	**39,951**	**30,968**	**29,453**^2^

^1^ Rtr = reverse transcriptase. Group refers to the different clusters of Fig 3.

^2^ The values in these lines are not the sum of the previous numbers because two different *in silico* “probes” can match within the distance range and considered as the same hit.

A further analysis was carried out in order to estimate the frequency of the putative incomplete RTs. The most frequent RT elements in lentil were used: *Angela*, *SIRE*, *Peabody* and *Ogre*. We searched for two LTRs of the same RT family within a distance of 10,000 bp, then for the presence of an internal sequence within of the LTRs. An internal sequence was identified in approximately only 3% of the *Angela* and *SIRE* (62 out of 1,767, and 59 out of 1,957) *Ty1-copia* elements, 28% (834/2,994) of the *Peabody* and 10% of the *Ogre Ty3-gypsy* elements (for *Ogre* a 50,000 bp distance was considered, since complete *Ogre* elements are larger than 10,000 bp).

**Fig 4:**
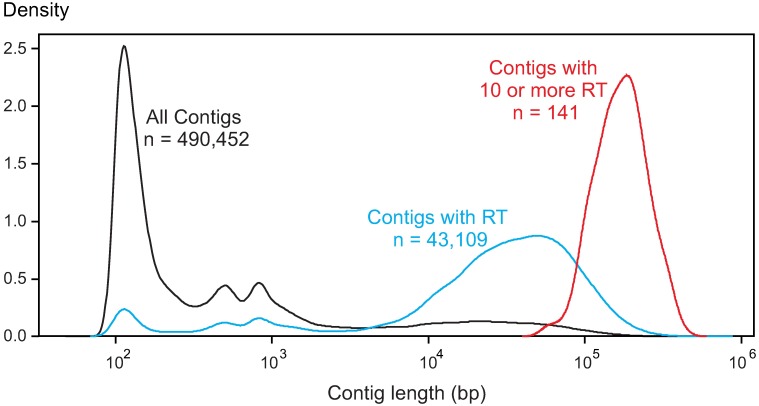
Contig length (bp) density distribution. Figure shows the distributions of contig lengths; blue and red color lines indicate contigs with at least one lentil LTR-RT sequence or 10 or more lentil LTR-RT, respectively. The black line indicates the distribution of all lentil contigs, V0.8 genome. n = number of contigs.

**Fig 5:**
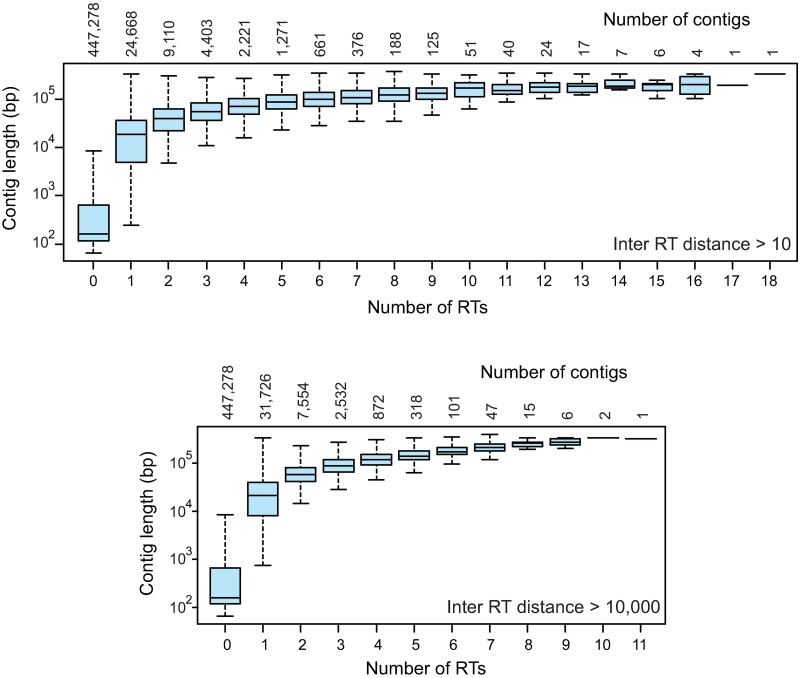
Boxplot distributions of contig length according to the number of “hits” generated by the lentil LTR-RT sequences. Numbers at the bottom indicate the number of hits per contig while those on top to the number of contigs in each class. The first distribution was obtained when two hits were considered different if they were separated by at least 10 bp, the second distribution when hits were separated by at least 10,000 bp.

Retrotransposon hit density distribution versus the logarithm of the contig length is in Fig 4. A total of 43,109 contigs (< 10%) contained one or more sequences similar to the “probes”, and most of them are large contigs (on average 42,770 bp). Only 141 of the largest contigs (mean size 176,200 bp) contained 10 or more sequences spaced by more than 10 bp. The distribution of the contigs with 0 to 18 LTR-RTs is depicted in Fig 5 with the box-plots indicating the average contig length and quartiles of each class. These results suggest that retrotransposons would be distributed randomly and that their number is directly related to contig length; thus the number included in each contig could possibly fit a Poisson distribution.

**Fig 6:**
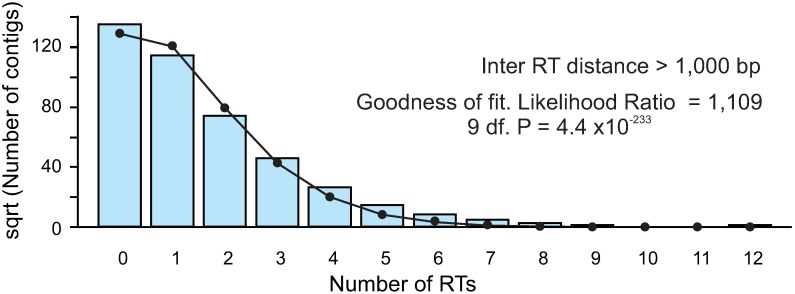
Goodness of fit testing a Poisson distribution of the LTR-RT number according to the square root of the number of contigs. The continuous line indicates the theoretical distribution and bars the real number of contigs within of each class. A between hit distance of > 10,000 bp was considered.

To test these hypotheses a regression analysis of the Poisson distribution was carried out using the general lineal model (glm). The natural logarithm of the number of elements in each contig was the dependent variable while the contig length in bp was the independent variable. The output models were similar with respect to the four between hit distances tested, as can be seen in S2 Table. The distance to consider two consecutive hits as belonging to different elements had only a minor effect on the coefficients. In all instances, results did not fit a Poisson distribution since the deviation of the residuals did not follow a normal distribution (S2 Table). Contigs evidencing a high deviation from the expected value consisted of some large contigs with a low number of elements. Deviations from the expected values could be due to the high number of contigs without hits, as well as to differences in contig length, therefore the fitting of the contig set with a size ranging 12,800 to 60,480 bp was tested (this set includes the intermediate size interquartile range excluding most of the contigs lacking hits). Again, data did not fit a Poisson distribution for any range of between hit distances. For instance, for a distance of more than 1,000 bp between hits an excess of contigs with no retroelements or else with four or more was observed, while a clear deficit in the classes with one or two elements was detected (Fig 6).

**Fig 7:**
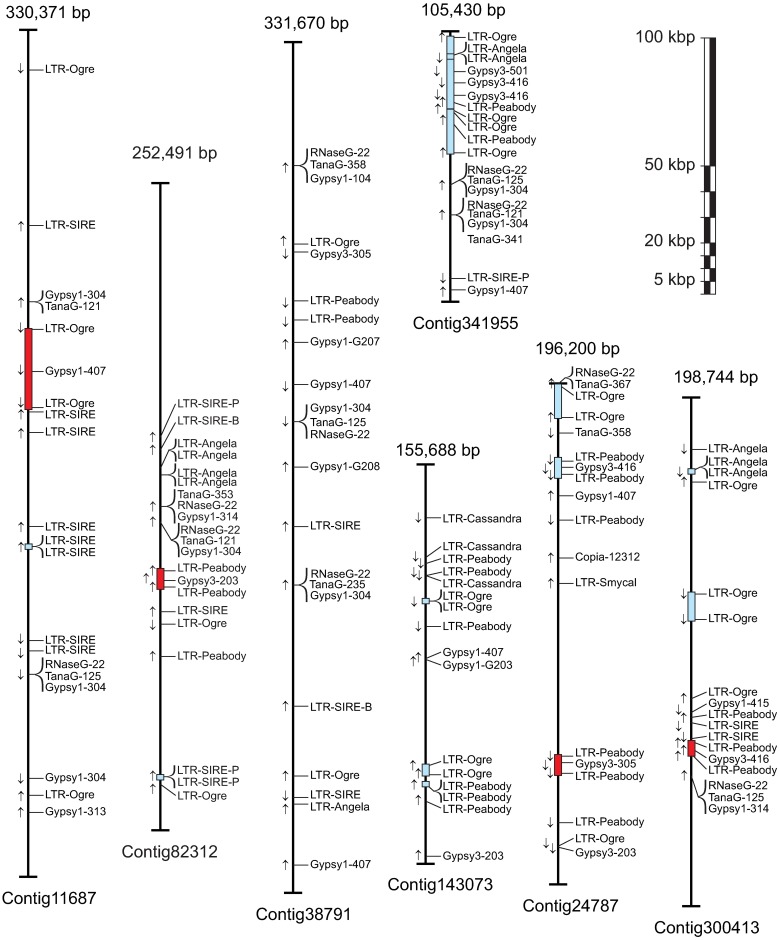
Diagram of the contigs in the lentil genome containing the highest number of LTR-RT sequences. Contig size (above) and contig name (below) are indicated. Arrows indicate sequences’ orientations. Blue boxes indicate putative RT flanked by two LTRs, red boxes indicate the presence of a reverse transcriptase sequence between LTRs. LTR are named according to their lineage (Table 2) and the internal sequences according to the nomenclature used in Table 1.

The physical maps of the possible retroelement locations corresponding to the seven contings accounting for the highest number of hits are shown in Fig 7. Only six possible complete LTR-RT were detected, two large *Ogre*-like (included in the *gypsy* family) in contigs 11687 and 341955, and four *Peabody*-like (also included in *gypsy*) with one in contigs 82312 and 300413 while two in contig 24787. The *Ogre*-like sequence of contig 11687 exhibited an internal hit for a reverse transcriptase of group 1e displayed in Fig 3B; while the sequence of contig 341955 probably constitutes a chimeric non-functional sequence since it included several internal hits of LTRs derived from other elements such as *Peabody* (*gypsy*) and *Angela* (*copia*) plus a reverse transcriptase (*gypsy* Group 3b). The four *Peabody*-like elements detected showed an internal hit of the *gypsy* reverse transcriptase belonging to Group 3b.

## Discussion

In this study, several retrotransposon-based markers such as iPBS and REMAP, were tested in relation to their utility to carry out genetic mapping in lentil. The iPBS technique [17] proved to be especially useful because it yielded a high number of Mendelian markers. Likewise, it provided a simple method to obtain LTR retrotransposon (LTR-RTs) sequences in order to gain knowledge on their type and distribution in plant genomes. Since the introduction of iPBS markers, they have extensively been used in plants with respect to fingerprinting and genetic diversity analyses [21–23, 50], nonetheless, their direct use in genetic mapping remains scarce. To the best of our knowledge, this study would be the first example of direct iPBS marker use in genetic mapping.

Considering the lentil parental lines used in this study, the high number of iPBS bands amplified in each reaction (an average of 9.75) indicates that LTR-RTs are abundant and widespread throughout of the lentil genome, in particular if considered that the iPBS PCR fragments are only produced when two LTR-RTs are located relatively close to each other and display inverted orientations. The high iPBS marker polymorphism detected in lentil agrees with previous results in pea [23]. Upon comparing the number of polymorphic iPBS loci (presence vs. absence of an amplified fragment) among the cultivated lentil and its wild ancestor, cultivar Lupa generated a slightly higher (106) number than that retrieved in the wild *L*. *orientalis* (87), although the difference was non-significant (χ^2^ = 1.87, 1 d.f., 0.2 > P > 0.1).

Segregating iPBS and REMAP markers were arranged in eight linkage groups, one more than the corresponding haploid chromosome set number of *Lens*. The marker density within each linkage group was uneven, evidencing in general a tighter clustering within of the central regions, possibly labeling favorable genomic regions for LTR-RT accumulation. Although a wide variation exists depending upon the specific LTR-RT element, in general LTR-RTs in other species tend to concentrate in gene-poor regions [51]. However, in *Medicago* and *Lotus*, LTR-RT rich regions can be as large as entire chromosome arms [52]. Analyzing the origin and distribution of the PCR product alleles, it can be observed (Fig 2) that in general these alleles derived equally from both parents and were distributed at random; yet in some linkage regions the alleles of one parental were clustered, such as in the case of linkage groups 1 and 6 exhibiting clusters of Lupa alleles. These regions might represent zones where the LTR-RT amplifications preferentially occurred, or LTR-RTs were inadvertently selected by linkage drag, in the cultivated lentil during the domestication process.

Sequencing of iPBS products and other retrotransposon based markers yielded a total of 81 sequences. The phylogenetic analysis disclosed that *Lens* contains all of the main LTR-RT families and phylogenetic lineages described in legumes [9]. According to our results, the lineage *Tat/Ogre* would be the most abundant in lentil, although the observed frequency could be biased due to the PCR primers used and is likely to be an underestimation since the current assembled lentil genome still lacks a large part comprising essentially repeated sequences. However, the *Ogre* prevalent abundance is in accordance with previous data of phylogenetically close species to lentil [9, 14]

Up to 37% of the lentil LTR-RT internal sequences analyzed in this study presented premature stop codons (PSC) having either originated by nucleotide substitutions or by frameshift mutations or by both mechanisms. The affected translatable sequences corresponded mainly to reverse transcriptase, since most of the analyzed sequences corresponded to this enzyme, but premature stop codons were also observed in other sequences. Since only a relatively reduced part of the total translatable fraction was sequenced, more PSC could possibly be present. Plant genome analyses in general tend to reveal a large number of LTR retrotransposons that contain stop codons interrupting their ORFs, lacking one or more coding domains, or else both situations [53]. Therefore, as in other plant species, a high proportion of the LTR-RTs would consist of non-autonomous elements lacking the essential enzymatic functions to enable genomic movement. However, most of these inactive or non-autonomous elements are likely to be able to retrotranspose using the trans-factors synthesized by autonomous elements present elsewhere in the genome, thus accordingly generating large changes in genome size [54–57]. The subsequent *in silico* search of homologous sequences in the lentil draft genome yielded a lower number of hits when the more numerous internal transposon sequences (i.e., reverse transcriptase, RNase H, etc.,) were used compared to the LTR sequences, suggesting that many lentil retrotransposons contain internal deletions. Thus, they could be included into the non-autonomous set.

When the distance considered between consecutive LTR hits increased from 10 up to 10,000 bp, the theoretical size of whole or nearly-whole retrotransposons, the number of identified elements should have been reduced by approximately 50%. However, the observed diminution was 15.9% for the *copia* elements and 22.9% for the g*ypsy* elements when the distance increased. Thus, the higher number of elements identified by the LTR hits compared to the internal sequence hits, plus the high frequency of internal sequence absence between LTRs, indicate in lentil a significant presence of defective non-autonomous, solo-LTRs and probably TRIM elements. Macas et al. [9] already indicated that the number of LTRs in species of the tribe Vicieae (Fabeae) was higher than that of the corresponding whole LTR-RTs; the estimated proportion of incomplete elements amounted to 41.2%, and 37.5% within of the *gypsy Ogre* elements (the most frequent retroelements).

In lentil the *Ty1-copia*, *Ty3-gypsy* and TRIM family frequencies are respectively 17.6%, 78.7% and 0.03% of the LTR-RTs [9]. The different lineage estimations obtained by our *in silico* search agree with these figures. When each one of the LTR sequences obtained was analyzed by separate, and we considered that they belonged to the same RT if two consecutive hits were separated by less than 10,000 bp, then the percentages obtained corresponded to 25.7% (10,898 hits) *Ty1-copia*, 72.0% (30,559) *Ty3-gypsy* and 2.3% (998) TRIM sequences (Table 3). Our data also agrees with previous results [9] for the within family lineages, especially for the major families: *Tat/Ogre* (59.8% of the *Ty3-gypsy* vs 66.0% in Macas et al., 2015), *Tekay/DEL/ Peabody* (40.2% of the *Ty3-gypsy* vs. 26%), *SIRE1/Maximus* (79.8% of the *Ty1-copia* vs. 93%).

Retrotransposons are generally widely distributed along plant genomes [7] and can be randomly distributed such as in the case of maize [58], although there are reports of preferential distribution of at least some types of retrotransposons, such as TRIMs [59–60], and retrotransposon-derived markers in plant genomes [7, 61–62]. The lentil elements analyzed were widely distributed as suggested by the percentage (8.8%) of the lentil draft genome contigs which contained at least one element. If LTR-RT were distributed at random in the genome, it would be expected that contigs containing zero, one, two, etc. LTR-RT should fit a Poisson distribution. But our data has not validated this hypothesis, even when a correction by contig sizes was considered. For instance, there are more large contigs without LTR-RT than expected (Fig 6). Furthermore, the genetic map generated with the iPBS markers (Fig 2) exhibited regions of different marker densities. These results suggest that the distribution of these elements in lentil is nonrandom.

## Conclusions

To sum up, the present data points out that LTR-RTs are a suitable source of genetic markers in lentil and to their utility in genetic analysis and map construction. These markers can be useful for future genetic analyses, marker-assisted breeding, and phylogenetic studies. In lentil, the results indicate that a high proportion of the lentil retroelements have lost their autonomous transposition ability, either by point mutations and/or deletions. In fact, many of them seem to embody defective elements with internal deletions. Likewise, although they seem to be widely distributed throughout of the lentil genome their distribution is not completely random, being LTR-RTs more densely clustered in some regions of the *Lens* genome.

## Supporting information

S1 Fig Partial amino acid sequences of the reverse transcriptase encoded by the lentil *Ty1-copia* elements. Asterisks denote premature stop codons and question marks the absence of one or two nucleotides in the corresponding reading frame. Conserved motifs are located inside boxes, lentil sequences denoted by “Cop” followed by a number (see Table 1), Mtr denotes sequences of *Medicago truncatula* as numbered according to Wang and Liu [44] indicating the clade in which they are included according to Piednöel et al. [46].
(PDF)

S2 Fig Partial amino acid sequences of the reverse transcriptase encoded by the lentil *Ty3-gypsy* elements. Lentil sequences are denoted by “Gyp” followed by a number See heading of Supplementary Fig 1 for additional legends.
(PDF)

S3 Fig Partial amino acid sequences of the reverse transcriptase and RNaseH codified by *Ty1*-*copia* elements obtained with Tnana-derived primers. RNaseH indicates the starting point of this protein. See heading of Supplementary Fig 1 for legends.
(PDF)

S4 Fig Partial amino acid sequences of the RNaseH codified by *Ty3*-*gypsy* elements obtained with Tnana-derived primers. See heading of Supplementary Fig 1 for legends.
(PDF)

S1 Table List of primers used. (PDF)

S2 Table Coefficients (a and b) according to the general linear model (glm) testing a Poisson distribution, and values of the D statistics followed by probability according to the Kolmogorov-Smirnov test of residuals adjusting to a normal distribution. (PDF)
