# Supplementary material for: Obtaining retrotransposon sequences, analysis of their genomic distribution and use of retrotransposon-derived genetic markers in lentil (Lens culinaris Medik.)
Source: PLoS One. 2017 Apr 27;12(4):e0176728. doi: 10.1371/journal.pone.0176728 (PMC5407846; doi:10.1371/journal.pone.0176728)
Supplement: S1 Fig — Asterisks denote premature stop codons and question marks the absence of one or two nucleotides in the corresponding reading frame. Conserved motifs are located inside boxes, lentil sequences denoted by “Cop” followed by a number (see Table 1), Mtr denotes sequences of Medicago truncatula as numbered according to Wang and Liu [44] indicating the clade in which they are included according to Piednöel et al. [46]. (PDF) [file pone.0176728.s001.pdf]

S1 Fig

```
Cop-304-G1     QMDVKTAFLNGELDEEVYMKQPEGFVIKVQEH-KVCKLTKSLYGLKQALKQWHQKFNQVMLANGYKINESDKCI*SKF-INGE-GVMI*LYVDDM
Cop-306-G1     QMDVKTAFLNGELDEEVHMKQPEGFVIKGQEH-KVCKLTKSLYGLKQAPKQ*HQKFDQVMLANGYKINESDKCIYSKF-NNEK-GVMICLYVDDM
Cop-307-G1     QMDVKTAFLNGELDEEVYMKQPEVFVIKGQEH-KVCKLTKSLYGLKQALKQWHQKFDQVMLANGYKINESDKCIYSKF-INGK-GVMI*LYVDDM
Cop-311-G1     QMDVKTAFLNGDLDEEVYMKQPEGFVVKGQEH-KVCKLTKSLYGLKQAPKQ*HQKFDQVMLANGYKINESDKCIYSKF-ISGK-GVMICLYVDDM
Cop-316-G1     QMDVKTAFLNGELDEEVHMKQPEGFVIKGQEH-KVCKLTKSLYGLKQAPKQ*HQKFDQVMLANGYKINESDKCIYSKF-NNEK-GVMICLYVDDM
Cop-321-G1     QMDVKTTFLNGELDEDVYMKQPKGLVIKGQEH-KVCKLTKSLYGLKQAPKQWHQKFDQVMFANGYKINESDKCIYSKF-NNKK-GVMICLYVDDM
Mtr7.7-C2      QMDVKTAFLNGELDEEVYMKQPEGFVIKGQED-KVCKLTKSLYGLKQAPKQWHQKFDQVVLANGYIINESDKCIYSKF-QNGK-GVMICLYVDDM
Cop-301-G2     QMDVKIAFSNGDLEEEVYMKQPEGFVMPGNEH-KVCKLVKSLYGLKQAPKQWHQKFDEVVLSNGFILNQADKCVYSKFDTSGK-GVFICLYVDDM
Cop-315-G2     QMDVKTAFLNGDLEEEVYMKQPEGFVMPSNEH-KVCKLVKSLYGLKQAPKQWHQKFDEVVLSNGFILNQADKCVYSKFDTSGK-GVFICLYVDDI
Cop-318-G2     QMDVKTAFLNGDLEEEVYMKQPEGFVMPGNEH-KVCKLVKSLYGLKQAPKQWHQKFDEVVLSNGFILNQADKCVYSKFDTSGK-GVFICLYVDDM
Cop-320-G2     QMDVKTTFLNGDLEE*VYMKQPEGFVMPGNEH-KVCKLVESLYGLKQAPKQWHQKFNEVVLSNGFILNQADKCVYRKFDTSGK-GVFICLYVDDM
Mtr4.1-C2      QMDVKTAFLNGELEEEIYMDQPEGFVIHGQEN-KVCKLDKSLYGLKQAPKQWHEKFDNLMIENEFKVNESDKCIYSKY-ENNT-CTIICLYVDDL
Cop-305-G5     QMDVKTAFLNGNLLEDVYMTQPEGFDIPQDAQ-KICKLQRSIYGLKQASRSWNLRFDETVKQYGFIKNEDEPCVYKKVSGSM--IVFLVLYVDDI
Cop-308-G5     QMDVKTAFLNGNLLEDVYMTQPEGFDIPQDAQ-KICKLQRSIYGLKQAYRSWNLRFDETVKQYGFIKNEDEPCVYKKVSGSM--IVFLVLYVDDI
Cop-310-G5     QMDVKTAFLNGNLLEDVYMTQPEGFDIPQDAQ-KICKLQRSIYGLKQASRSWNLRFDETVKQYGFIKSEDEPCVYKKVSGSM--IVFLVLYVDDM
Cop-312-G5     QMDVKTAFLNGNLLEDVYMTQSEGFDIPQDAQ-KICKLQRSIYGLKQASRSWNLRFDETVKQYGFIKNEDEPCVYKKVSGSM--IVFLVLYVDDI
Cop-314-G3     QMDVKTTFLYGDLKEEI*ME*PKGYVLPGNEQ-KV*KLVKSLYGLKQAPK----?FDSVVLSDGFIPNSCDKCLYTKDW-K-F-LIFMCLYVDDM
Cop-317-G3     QMDVKTTFLKGDLEEEIYMEQLEGYVLPGNEQ-KVCKLVKSLYGLKQAPKQWHQKFDSYVLSNGFIPNSCDKFLYTKDC-KTT-VIFLCLYVDDM
Cop-322-G3     QMDVKTTFLNGDLDEEIYMEQPEGYVLPGNE*-KACKLVKSLYSLKHAPKQWH*NFESVIISNGFIPNSCGKCSYTKDW-K?I-IIFMCLYVDDM
Cop-323-G4     QMDVKTTFLNGGLEEEVYMKQPEGFSSNNGEH-LACKLKKSIYGLKQASRQWYLKFHGTISSFGFIENPMDQCI*PEGQWE*--ICFLILYVDDM
Mtr18.2-C3     QMDVKTAFLNGNLLEDVYMTQPEGFGDPKATK-KVCKLQRSIYGLKQASRSWNLRFDETVQQYGFIKNEDEPCVYKKVSGSI--VSFLILYVDDI
Cop-302-G6     QMDVKTAFLHGDLEEEIYMK*PDGFLDKGKED-YVCRLRKSLYGLK*APRQWYKKFESVMCENDYKKTTSDNCIFVKFFTNDD-IIILLLYVDDM
Cop-303-G6     QMDVKTAFLHGDLEEEIYMKQPDDFLAKGKED-YVCRLRKSLYGLKQAPRQWYEKFESVIYEQDYKKATSDHCVFIKFFTNDD-IIILLLYVDDM
Mtr16.4-C1     QLDVKTAFLHGELEEEIYMLQPEGFKEQGKEN-LVCRLTKSLYGLKQAPRCWYKRFDSFIISLDYSRLSSDHCTYYKRFDGND-FIILLLYVDDM
Cop-319-G7     QMDVKTTFLNGVLEEEVYVEQP?WYMKVGEEK-KVLKL?KALYGLKQAPRAWNTRIDTYFKDNGFRQCPYEHALYVKK-NQGN-LLLV?LYVDDM
Mtr48.2-C4     QLDVKSTFLNGELEEEVYVKQPQGFEVEGKEG-KVYKLHKALYGLKQAPRAWNSKIDAYFLQNGFVKSPSEPSLYVKR-SGAN-FLMVCLYVDDL
Mtr10.1-C4     HLDVKSAFLNGPLDEEVYVTQPPGFKIKGKED-MVYRLHKALYGLKQAPRAWNKRIDSFLVKQEFVKCKSEYGVYVKKGSEGN-QLLICLYVDDL
Mtr2.1-C5      QMDVKSAFLNGFLNEEVYVSQPPGFINKEKPN-HVFKLTKALYGLKQAPRAWYDRLSTFLIENGFSRGKIDTTLFRKTH-NTD-LLIVQVYVDDI
Mtr11.1-C7.1   QMDVKNAFLHGDLQEEVYIKLPNGMPTP-SPN-TVCKLKRSLYELKQAPRVWFEKFRSTLIGFEFSQSQYDPSLFLQRT-PKG-MVVLLVYVDDI
Mtr38.1-C7.2   QLDIKNAFLHGDLEEEVYMEQPPGFVAQGESSTMVCRLHRSLYGLKQSPQAWFGRFSTVVQQFGMIRSQADHSVFYRHS-PQG-CIYLIVYVDDI
Mtr20.1-C7.2   QLDVKNAFLNGDLQEEVYMDSPPGFEDKFGLN--VCKLQKSLYGLKQSPRAWFEKFTWSVKKQGYMQAQSDHTLFMRFSNDGK-IAILIVYVDDI
Mtr17.1-C6     QLDVNNAFLHGDLNEEVYMVLPPGLKLQNSDSNDLKVCRLNKSLYGLKQASRQWYAKLSAALVSLGYTPSVADSSLFTKLKGTNFTALLVYVDDI
Mtr9.1-C8      QLDVNNAFLNGVLTEEVYMVQPPGFESS-DKN-LVCKLHKALYGLKQAPRAWFERLKSSLLSFGFKSSRCDPSLFTLHT-QAY-CIFILVYVDDI
```
