# Supplementary material for: Obtaining retrotransposon sequences, analysis of their genomic distribution and use of retrotransposon-derived genetic markers in lentil (Lens culinaris Medik.)
Source: PLoS One. 2017 Apr 27;12(4):e0176728. doi: 10.1371/journal.pone.0176728 (PMC5407846; doi:10.1371/journal.pone.0176728)
Supplement: S2 Fig — Lentil sequences are denoted by “Gyp” followed by a number See heading of Supplementary Fig 1 for additional legends. (PDF) [file pone.0176728.s002.pdf]

S2 Fig

```
Gyp-409-G2      RMCVDYRKLNKATRNDHFPLPVIDQMLERL?KHSHFCYLDGYSGFFQIPIHHDDQ?KTTFTCPYGTFAYKRMPFGLCNAPANFQRCMM-IFADFLDNIMEVFMDNFSICGKSFEGCLSNLEMVLERCVKVNLVLNWEKFHF
Mtr80.2         RMCIDYRKLNKATRKDHFPLPFIDQMLERLAKHSHFCYLDGYSGFFQIPIHPNDQEKTTFTCPFGTFAYRRMPFGLCNAPATFQRCMMSIFSDFVEKIMEVFMDDFSVHGSNFDDCLTNLEKVLERCEQVNLVLNWEKCHF
Mtr60.1-Athila  RMCIDYRRLNQATRKDHFPLPFMDQMLERLSGQAYYCFLDGYSGFNQITVNPVDHEKTAFTCPFGIFAYRRMPFGLCNAPATFQRCMQAIFSDLIEKSIEVFMDDFSVFGSSYDVCLNNLDTVLKRCKETNLVLNWEKCHF
Gyp-201-G3      RMCVDYRQLNKVTIKNRYPLPRIDDLMDQLVGASVFSKIDLRSGYHQIRVNTEDIQKTAFRTRYGHYEYSVMPFGVTNAPGVFMEYMNRIFHPYLDMFVVVFIDDILVYSKSEEEHAEHLRAVLVPL
Gyp-203-G3      RMCVDYRQMNKVTIKNRYPLPRIDDLMDQLVGASVLSKIDLRSGYHQIRVKTEDIQKTAFKTRYGHYEY.
Gyp-305-G3      KMCVDYRQLNKVTIKNRYPLPRIDDLMDQLVDASVFSKIDLRSGYHQIRVKTKDIQKTAFRTRYGHYEYSVMPFGVTNAPGVFMEYMNRIFHPYLDKFVVVFIDDILVYSKSEEEHAEHLRVVLEVLREKKLFAKLSKC
Gyp-408-G3      KMCVDYRQLNKVTIKNKYPLPRIDDLMDQLIGASVFSKIDLRSGYHQIKVKDEDV*KTTFRTRYGHYEYFVMPFGVTNAPGIFMEYMNRIFHEYLDRFVVVFIDDILVYSKSVAEHAEHLRIVLEVLKERKLYAKLSKC
Gyp-416-G3      RMCVDYRQLNKVTIKNRYPLLRIDDLMDHLVGASVFSKIYLRSGYHQIRVKTEDIHKTAFRTRYGHYEYFVISFGVTNAPGVFMEYMNRIFHPYLDKFVVVFIDDILIYSKNEEEHVEHLRMVFEVLREKKLFAKLSKS
Gyp-501-G3      RMCVDYRQLNKVTIKNQYPLPRIDDLMDQLVGASVFSKIDLRSGYHQIRVKTEDIQKNALRTRYGHYEYFVMPFGVTNCNTLIFTPI?NTHI
Gyp-512-G3      RMCVDYRQLNKVTIKNQYPLPRIDDLMDQLVGASIFSKIDLMCGYHQIRVKAEDIQKTAFRTRYGHYEY
Mtr63.2-Tekay   RLCIDYRQLNKVTIKNRYPLPRIDDLMDQLVGAKIFSKIDLRSGYHQIKVKDEDMQKTAFRTRYGHYEYKVMPFGVTNAPGVFMEYMNRIFHAYLDKFVVVFIDDILIYSRTEEEHAEHLRVVLQVLKEKKLYAKLSKC
Mtr69.1-Tekay   RLCIDYRQLNKVTIKNRYPLPRIDDLMDQLVGAKIFSKIDLRSGYHQIKVKDEDMQKTAFRTRYGHYEYKVMPFGVTNAPGVFMEYMNRIFHAFLDKFVVVFIDDILIYSKNEEEHAEHLRIVLQVLKEKRLYAKLSKC
Mtr58.2-Tekay   RLCIYYRQLNKVTIKNRYPLPRIDDLMDQLVGASVFNKIDLRSGHHQIKVKDEDM*KTTFRTRYGHYEYKVMPFGVTNALGVFMEYMNRIFHAFLDRFVVVFIDDILIYSKSEEEHAEHLKLVLQVLKKKKLYAKLSKC
Mtr72.1-Reina   RFCVDYRALNALTVKDRFPIPAIDELLDELHGTRWFSKLDLRSGYHQIRMAPQDTHKTAFRTHQGHYEFLVMPFGLSNAPSTFQSSMNRILQPYLRQFVIVFFDDILIYSPTLEDHRHHLEVVFNCLLENQFCLKYSKC
Mtr65.1-CRM     RMCVDSRAVNKITIKYRFLIPRLDDLLDQLHGATIFSKIDLRSGYHQIRIRPGDEWKTAFKTRDGLYEWTVMPFGLSNAPSTFMRLMNQILRPFIDKFIVVYFDDILIYSKHKEEHLEQLRQVLQTLREQKLYANLKKC
Gyp-104-G1a     SMCVDYRDLNRASPKDDFLLPHIEVLVDNTTHAKVFSFMDGFSGYNQIKMAPEDMEKTAFITPWGTFYYKVMPFGLKNAGATYQRVMVTLFHDMIHKEIEVYVDDMIAKSQTEEEHLVNLQKLFKRLRKFQLRLNPSKCTF
Gyp-314-G1a     KMCVD?QDLNRASPKDDFMLPHIDVLVDNTAHAKVFSFMDGFSGYNQIKMAPEDMEKITFITPWGTFCYKVMPFGLKNAGATYQ
Gyp-307-G1a     HMCVDYRDLNRVSPKDDFMLPHIDVLVDNTAHAKVFSFMDGFSGYNQIKMAPEDMEKIAFITPWGTFCYKVMPFGLKNAGATYQ
Gyp-402-G1a     RMCVDYRDLNRAIPKDDFSLPHIDVLVDNIAHAKVFSFMDGFSGYNQIKMASYDMEKTAFITPWGTFCYKVMPFGLKNAGATYQHAMV
Gyp-304-G1a     RMCVDYRDLNRASPKDDFLLPHIEVLVDNTTHAKVFSFMDGFSGYNQIKMAPEDMEKTAFITPWGTFYYKVMPFGLKNAGATYQRVMVTLFHDMIHKEIEVYIDDMIAKSQTKEEHLVNLQKLFERLRKFQLRLNPNKCTF
Gyp-306-G1b     KMCVDYRDLNKASPKDDFPLPHIDVMVDNTAQHKVFSFMDGFSGYNQIKMAPEDMEKTTFVTQWGTFCYKVMPFGLKNAGGNIQRAMVVLFHDMIHHEIEVYVDDMIARSQTEEEHLDHLYKLFERLKKYKLRLNPNKC
Gyp-401-G1b     RMCVDYRDLNKASPKDDFPLPHIDVMVDNTAQHKVFSFMDGFSGYNQIKMAPEDMEKTTFVTQWGTFCYKVMPFGLKNAGATYQRAMVVLFHDMIHHEIEVYVDDMIARSQTEEEHLDHLYKLFERLKKYKLRLNPNKC
Gyp-105-G1c     SMCVDYRDLNKASLKDDFPLPHIDVLVDNTAQYSVFSFMDGFSGYNQIKMSLEDMEKTTFITPWGTFCYKVMPFGLKNAGATYQRAMVTLFHDMIHKEIEVYVDDMIAKSQTEEVHLVHLKKLFERLREFKLRLNPNKCTF
Gyp-212-G1c                         ?YIDVLVDNTAQFSVFSFMDGFSGYNQIKMSPEDMEKTTFITPWGTFCYQVMPFDLKNAGETYQRAMVTLFHDMMHKEVEVYVDDM
Gyp-313-G1c     SMCVDYRDLNKASLKDDFPLPHIDVLVDNTAQFSVFSFMDGFSGYNQIKMSLEDMEKTTFITPWGTFCYKVMPFGLKNAGATYQRAMVTLFHDMIHKEIEVYVDDMIAKSQTEEVHLVHLKKLFERLREFKLRLNPNKCTF
Gyp-412-G1d     RMCVDYRDLNRASPKDDFPLPHIDMLVDNTTKFSVFSCMDGLSGYNQIKMAPEDMEKTTFITPWGTFCYRVMPFGLKNVGATYQRAMTTLFHGMMHKEIEVYVDDMIAKSHSEEDHL
Gyp-201-G1e     RMCVDYRDLNRASPKDDFPLPHIDTLVDNTAKFDIFSFMDGFSGYNQIKTAPEDMEKTTFITPWGTFCYQVMPFGLKNAGATYQRAMTMLFHDMMHKEIEVYVDDM
Gyp-203-G1e     RMCVDYRDLNRASPKDDFPLPHIDMLVDNTTKFDIFSFMDGFSGYNQIKMAPEDMENTTFITPWGTFCYQVMPFGLKNAGATYQRAMTTLFHDMMHKEIKVYVDDM
Gyp-207-G1e     RMCVDYRDLNRASPKDDFPLPHIDMLVDNTTKFDIFSFMDGSSGYNQIKMAPEDMEKTTFITPWGTFCYQVMPFGLKNAGATYQRAMTTLFHDMMHKEIEVYVDDM
Gyp-208-G1e     RMCVDYRDLNRASPKDDFSLPHIDMLVDNTAKFDIFSFMDGFSGYNQIKMAPEDMDKTTFITPWGTFCYQVMPFGLKNAGATYQRAMTTLFHDMMHKEI*VYVDDM
Gyp-210-G1e     RMCVDYSDLNRASPNDDFPLPHIDMWVDNTTKFDIFSFMDGFSGYNQIKMAPEDMEKTTFITPWGTFCYQVMPFGLKNAGATYQRAMTMLFHDMMHKEIEVYVDDM
Gyp-407-G1e     RMCVDYRDLNKASPKDDFP?PHIDMLVDNTTKFDIFSFMDGFSGYNQIKMAPEDMEKTTFITPWGTFCYQVMPFGLKNACAAYQRAMTMLFHDMMHKEIEVYVDDMIAKSRSEEGHLVDLLKLFQRLRKFCLRLNPNKCTF
Gyp-308-G1d      MCVDYRDLNRSIPKDDFPLPHIDMLVDNTAKFSIFSFMDGFSGYNQIKMAPEDMEKTTFITPWGTFCYKVMPFGLKNAGATYQRAMVTLFHDMIHKEIEVYVDDMIAKSQTEEVHLVHLKKLFERLREFKLRLNPNKCTF
Gyp-405-G1d     NMCVD?QDLNRASPKDDFPLPHIDMFVDNTTKFSVFSFMDGFSGYNQIKMAPEDMEKTTFITPW?TFCYRVMPFGLKNAGAT*QRAMTTLFHDMMHKEIKVYVDDMIAKSHSEEGHLIDLLKLFQRLRKLRLRLNPNKCTF
Gyp-415-G1d     RMCVDYRDLNRASPKDDFPLPHIDMFVDNTTKFSVFSFMDGFSGYNQIRMAPEDMEKTTFITPWGTFCYRVMPFGLKNAGAT*QRAMTTLFHDTMHKEIKVYVDDMIAKSHSEEGHLIDLLKLFQRLRKFRLRLNPNKCTF
Mtr70.1-Tat     RMCVDYRDLNKASPKDDFPLPHIDVLVDSTAKSKVFSFMDGSSGYNQIKMAPEDREKTSFITPWGTFCYRVMPFGLINAGATYQRGMTTIFHDMIHKEIEVYVDDMIVKSITEEQHVEYLLKMFQRLRKYRLRLNPNKCTF
Mtr59.19-Tat    RMCVDYRDLNKASPKDDFPLPHIDVLVDSTARCKVFSFMDGFSGYNQIKMAPEDREKTSFITPWGAFCYLVMPFGLINAGATYQRGMTKIFHDMIHKEIEVYVDDMIVKSGTEEEHVEYLLKMFHRLRKYKLRLNPNKCTF
Mtr57.42-Tat    RMCVDFRDLNKASPKDNFPLPHIDVLVDNTAQSKVFSFMDGFSGYNQIKMSPEDREKTSFITPWGTFCYKVMPFGLINAGATYQRGMTTLFHDMIHKEVEVYVDDMIVKSTDEEQHVEYLTKMFERLRKYKLRLNPNKCTF

Group 1 (continue)                                                                                                                  RNaseH Start
Gyp-104-G1a   GVQSGKLFGFIVSHRGIEVDPAKIKAIQEMPFPETEKQVRGFLGRLNYIASFISHLTYTCEPLFKFLRKDQAIVWNDDCHQAFDKIKEYLQESPILMPPVPGRPLIMYLTVLAHSMGCVLWQPDKSCRKCH
Gyp-304-G1a   GVQSGKLFGFIVSHRGIEVDPAKVKAIQEMPFPETEKQVRGFLGRLNYIASFISHLTSTCEPLFKFLRKDQAIVWNDDCQQAFDKIKEYLQESPILMPPVPGRPLIMYLTVLDNSMGCVLWQHDESGRKCH
Gyp-105-G1c   GVRSGKLFGFIVSQRGIEVDPDKVKAIQNMPPPRTEKEVR
Gyp-313-G1c   GVRSGKLFGFIVSQRGIEVDPDKVKAIQNMPPPRTEKEVR
Gyp-308-G1d   GVRSGKLFGFIVSQRGIEVDPDKVKAIQNMPPPRTEKEVR
Gyp-405-G1d   GVRSGKLLGFVVSQKGIEVDPDKVRAIQEMPAPKTEKQVRGFLGRLNYISRFISHMTATCEPTF?LLKKSQSCVWTADFQKAFDSIKEYLLEPPILLPPVEGRPLIVYLTVLETSMGCILGQQDETGRKEYAIYYLSKKFTDCESR
Gyp-415-G1d   GVRSGKLLGFVVSQKGIEVDPDKVRAIQEMPAPKTEKQVRGFLGRLNYISRFISHMTATCEPTFKLLKKSQSCVWTADFQKAFDSIKEYLLEPPILLPPVEGRPLIMYLTVLETSMGCILGQQDETGRKEYAIYYLSKKFTDCESR
Gyp-407-G1e   GVRSGKLLGFIVSQKGIEVDPDKVKAIQEMPAPKTEKKVRGFLGRLNYISRFISHMTATCEPIFNF
Mtr70.1-Tat   GVRSGKLLGFIVSQKGIEVDPDKVKAIREMPAPQTEKQVRGFLGRLNYISRFISHMTATCGPIFRLLRKDQGIVWTEDCQKAFDSIKVYLLEPPILIPPVEGRPLIMYLTVLEDSMGCVLGQQDETGRKEHAIYYLSKKFTDCESR
Mtr59.19      GVRSGKLLGFIVSQKGIEVDPDKVKAIREMPAPRTEKQVRGFLGRLNYISRFISHMTATCGPIFKLLRKDQGIVWTEDCQKAFDSIKGYLLEPPILVPPVEGRPLIMYLTVLEDSMGCVLGQQDETGKKEHAIYYLSKKFTDCESR
Mtr57.42      GVRSGKLLGFIVSQKGIEVDPNKVRAIREMPAPQTEKQVRGFLGRLNYISRFISHMTATCGPIFKLLRKNQPIVWNDECQEAFDSIKNYLLEPPILVPPVEGRPLIMYLAVFDESMGCVLGQQDETGKKEHAIYYLSKKFTDCETR
                                                                                                               TNanaG Start
```
