# Supplementary material for: Obtaining retrotransposon sequences, analysis of their genomic distribution and use of retrotransposon-derived genetic markers in lentil (Lens culinaris Medik.)
Source: PLoS One. 2017 Apr 27;12(4):e0176728. doi: 10.1371/journal.pone.0176728 (PMC5407846; doi:10.1371/journal.pone.0176728)
Supplement: S3 Fig — RNaseH indicates the starting point of this protein. See heading of Supplementary Fig 1 for legends. (PDF) [file pone.0176728.s003.pdf]

S3 Fig

```
                                                                     RNaseH
Tnana-343  ----------------------------KGLSLLFTYPTTIPRISRCYLLRVCSY---FESNQVTPEPLQSKDGLVISQRKYAMDILEETGLLNAKPADTPMDPSVKLLPNQGEPLSDPGRYRRLVGKLNYLT
Tnana-345  ----------------------------KGLSLLFTYPTTIPRISRCYLLRVCSY---FESNQVTPEPLQSKDGLVISQRKYAMDILEETGLLNAKPADTPMDPSVKLLPNQGEPLSDPGRYRRLVGKLNYLT
Tnana-352  ----------------------------KGLSLLFTYPTTIPRISRCYLLRVCSY---FESNQVTPEPLQSKDGLVISQRKYAMDILEETGLLNAKPADTPMDPSVKLLPNQGEPLSDPGRYRRLVGKLNYLT
Tnana-356  ----------------------------KGLSLLFTYPTTIPRISRCYLLRVCSY---FESNQVTPEPLQSKDGLVISQRKYAMDILEETGLLNAKPADTPMDPSVKLLPNQGEPLSDPGRYRRLVGKLNYLT
Tnana-357  ----------------------------KGLSLLFTYPTTIPRISRCYLLRVCSY---FESNQVTPEPLQSKDGLVISQRKYAMDILEETGLLNAKPADTPMDPSVKLLPNQGEPLSDPGRYRRLVGKLNYLT
Tnana-359  ----------------------------KELSLLFTYPTTIPRISRCYLLRVCSY---FESNQVTPEPLQSKDGLVISQRKYAMDILEETGLLNAKPADTPMDPSVKLLPNQGEPLSDPGRYRRLVGKLNYLT
Tnana-245  --YTISHLDLINYQLAH*SVSFLVIIEPKGLSLLFTYPTTIPRISRCYLLRVCSY---FESNQVTPEPLRSKDGLVISQRKYAMDILEETGLLNAKPADTPMDPSVKLLPNQGEPLSDPGRYRRLVGKLNYLT
Tnana-244  --YTIFLLDLINYQLAH*SVSFLVIIDPKGLSLLFTYPTTIPRISRCYLLRVCSY---FESNQVTPEPLQSKDGLVISQRKYAMDILEETGLLNAKPADTPMDPSVKLLPNQGEPLSDPGRYRRLVGKLNYLT
Tnana-242  --YTIFLLDLINYQLAH*SVSFLVIIDPKGLSLLFTYPTTIPRVSRCYLLRVCSY---FESNQVTPEPLQSKDGLVISQRKYAMDILEETGLLNAKPADTPMDPSVKLLPNQGEPLSDPGRYRRLVGKLNYLT
Tnana-241  --YTIFLLDLINYQLAH*SVSFLVIIDPKGLSLLFTYPTTIPRISRCYLLRVCSY---FESNQVTPEPLQSKDGLVISQRKYAMDILEETGLLNAKPADTPMDPSVKLLPNQGEPLSDPGRYRRLVGKLNYLT
Tnana-112  PLLCHDLLLFSFIVTQ?PKGY*SYC?YVDDIVITGSNQQGILQLKQHLSN*L*KKDFGKL*YFLGI*VAQSKDGLMISQRKNAMDILE?TGLLNAKPADTPMDPSVKVLLNQGEPLSDSGRYRRLVGKLNYLT
Tnana-312  -----------FIVTQ-LNGYLSYW-Y-RDIVITGSDQQGILRLKQHLSNQFQTKDLGKLRYFLGIEVAQSKYGLVISQRKYAMDILEETRLLNAKPADTPMDPSVKLLSNQGEPLSDQGRYRRLVGKLNYFT
Tnana-311  -----------FIVTQ-LNGYLSYW-Y-RDIVITGSDQQGILQ-*KHLSNQFQTKDLGKLRYFLGIEVAQSKDGLVISQRKYAMDILEETCLLNDKPADTPMNPSVKLLPNQGEHISDSVRYMRLVGKLNYLT
Tnana-224  -----------------------------------------------------------------------------------------------------------------------------------VH
Tnana-334  ----------------------------------GSDQQEILQLKHISRINFRQKIFVNSATSWELRWLNPNMVW*-FPNGNMLWIFWKKQVVECKPTDTLMDLSVKLLPNQGEPLSDQGRYRRLVGKLNYLT


Tnana-343  VTRPDISFAVSVVSQFLNSPCQEHMDAVIRILRYIKSAPGKGLVYEDKGHTQIVGYSDADWAGSPIDRQSTSGYCVLVGGNLISWKSKKQNVVARSSAEAEYRVLALVTCE
Tnana-345  VTRPDISFAVSVVSQFLNSPCQEHMDAVIRILRYIKSAPGKGLVYEDKGHTQIVGYSDADWAGSPIDRQSTSGYCVLVGGNLISWKSKKQNVVARSSAEAEYRVLALVTCE
Tnana-352  VTRPDISFAVSVVSQFLDSPCQEHMDAVIRILRYIKSAPGKGLVYEDKGHTQIVGYSDADWAGSPIDRQSTSGYCVLVGGNLISWKSKKQNVVARSSAEAEYRVLALVTCE
Tnana-356  VTRPDISFAVSVVSQFLNSPCQEHMDAVIRILRYIKSAPGKGLVYEDKGHTQIVGYSDADWAGSPIDRQSTSGYCVLVGGNLISWKSKKQNVVARSSAEAEYRVLALVTCE
Tnana-357  VTRPDISFAVSVVSQFLNSPCQEHMDAVIRILRYIKSAPGKGLVYEDKGHTQIVGYSDADWAGSPIDRQSTSGYCVLVGGNLISWKSKKQNVVARSSAEAEYRVMAIATCE
Tnana-359  VTRPDISFAVSVVSQFLNSPCQEHMDAVIRILRYIKSAPGKGLVCEDKGHTQIVGYSDADWAGSPIDRQSTSGYCVLVGGNLISWKSKKQNVVARSSAEAEYRVMAIATCE
Tnana-245  VTRPDISFAVSVVSQFLNSPCQEHMDAVIRILRYIKSAPGKGLVYEDKGHTQIVGYSDADWAGSPIDRQSTSGYCVLVGGNLISWKSKKQNVVARSSAEAEYRVMALVTCE
Tnana-244  VTRPDISFAVSVVSQFLNSPCQEHMDAVIRILRYIKSAPGKGLVYEDKGHTQIVGYSDADWAGSPIDRQSTSGYCVLVGGNLISWKSKKQNVVARSSAEAEYRVMAMATCE
Tnana-242  VTRPDISFAVSVVSQFLNSPCQEHMDAVIRILRYIKSAPGKGLVYEDKGHTQIVGYSDADWAGSPIDRQSTSGYCVLVGGNLISWKSKKQNVVARSSAVAEYRVMALVTCE
Tnana-241  VTRPDISFAVSVVSQFLNSPCQEHMDAVTRILRYIKSAPGKGLVYEDKGHTQIVGYSDADWAGSPIDRQSTSGYCVLVGGNLISWKSKKQNVVARSSAEAEYRVMAMATCE
Tnana-112  VTRLDFSFAVSVVSHFLNSPCQEHMDVAIRILRYIKYAPGEGPVYDDKGHTQIVGYS--DWAGSPIVRRSTSGCCVLVGGNHIAWKSKKQNEVARSRVEAEYRVMAIVTCE
Tnana-312  VTRPDISFAVSVVSQFLNSLCQEHMDAVIRILRYIKCAPGKGLVYKNKRHTP?-----VDWARSPIDRRSTSGYCVLVGGNLISWKSKKQNVVARLSAEAEYRVMAIVTCE
Tnana-311  VTRPDISFVVSVVSQFLSSPCQEHMDVVIQILRYIKSAPRKGLVYGDKGHTQIVGYSDVERAGSPIDRRSISGDCVLVGGNLISWKS*KQNEVARSNNEVEYRFMALVTCE
Tnana-224  NLSPGISFAVSVVSQFLNSPCQEHMDAAIRILSYIKCAPGKGLVYEIKGHTQIVGYSDADWAGSPIDRRSTSGYCVLVGGNLIS*KSKK*NVVARSSAEAEYRAMALATCE
Tnana-334  VTCPDISFAVSVVSQFLNSPCQEHMDVVIRILRYIKCAPGKGLVYDNKGHTQMDRYSDADWAGSPIDRQSTSGYCVLVGGNLISWKSKKQNVVARSSVEAKYRVMALVTCE
```
