# Supplementary material for: Obtaining retrotransposon sequences, analysis of their genomic distribution and use of retrotransposon-derived genetic markers in lentil (Lens culinaris Medik.)
Source: PLoS One. 2017 Apr 27;12(4):e0176728. doi: 10.1371/journal.pone.0176728 (PMC5407846; doi:10.1371/journal.pone.0176728)
Supplement: S4 Fig — See heading of Supplementary Fig 1 for legends. (PDF) [file pone.0176728.s004.pdf]

S4 Fig

```
Tnana-121  VHMLLVPGR-PLIMYLTVLDNSMGCVLGQHDESGRKEYVIYYLSKKFTDCETRYSLLERTCCALVWAARRLRQYMLAHTTLLISRMDPVKYIFEKPALTGRVARWQMILTEYDIQYVTQKAIKGSV
Tnana-358  VHRLLLPGR-PLIMYLTVLDKSMGCVLGQHDESGRKEYAIYYLSKKFPDCETRYSLLERTCCALVWAARRLRQYMLAHTTLLISRMDPVKYIFEKPALTGRVARWRMILTEYDIQYVTQKAIKGSI
Tnana-367  VHMLLLPGR-PLIMYLTVLDNSMGCVLGKHDDSGRKEYAIYYLSKKFTDCETRYSLLERTCCALVWAARRLRQYMLDHTTLLISRMDPVKYIFEKPALTGRVARW*MILTEYDIQDST--------
Tnana-353  VHMLPLPGG-PLIMYLTVLDNSMGCVLGQHDESGRKEYAIYYLSKKFTDCETRYSLLERTCCALVWAARRLRQYMLAHTTLLISRMGPVKYIFEKPALTGRVARWQMILTEYDIQYVTQKAIKGSV
Tnana-235  VHMLPLPGRDPLIMYLTVLDNSNGGV?GQHDESGRKEYAIYYLSKKFTDCETRYSLLERTCCALVWAARRLRQYMLAHTTLLISRMDPVKYIFEKPALTGRVARWQMILTEYDIQYVTQKAIKGSV
Tnana-341  VHMLPLPER-PLIMYLTVLDNSMGCVLGQHDEFGRKEYAIYYLSKKFTDCET*YSLLERTCCALVWAARRLR*YMLAHTTLLISRMDPVKYIFEKPALTGRVARWQIILTEYDIQYVT*KAIKGSV
Tnana-123  VHMLLVPGR-PLIMYLTVLDNSMGCVLGQHDESGRKEYVIYYLSKKFTDCETRYSLLERTCCALVWAARRLRQYMLAHTTLLISRMDPVKYIFEKPALTGRVARWQMILTEYDIQYVTQKAIKGSV
Tnana-125  VHMLPLPGR-PLVMYLTVLDNSMGCVLGQHDESGRKEYAIYYLSKNFTDCETRYSLLERTCCALVWAARRLRQYMLAHTTLLISRMDPVKYIFEKPALTGRVARWQMILMEYDIQYVTQKAIKGSV

Tnana-121  LSDYLAHQPVDDYQPMKFEFPDEDIMHCETPVLEEGPEPGARWTLVFDGASNSLGHGIGAVITSPAGFHIPFTARLCFDCTNNTTEYEACIFGLEAAIDLKIKFLEVYGDSAMVTCE
Tnana-358  LSDYLAH*PVDDYQPMKFEFQDEDIMNCGIPVTEEGPEPGARWTLVFDGASNSLGHGIGAVITSPAGFHIPFTARLCFDCTNNTAEYEACIFGLEAAIDLKI?FHEVCGDSAMATCE
Tnana-367  -----------------------------------------------------LGHGIAAVI?SPAGFHIPFTARLCFDCTTNTAEYEACIFGLEAAIDLKIKFLVVYGDSALATCE
Tnana-353  LSDYLAHQPVDDYQPMKFEFPDEDIMHCETLVLEEGPEPGARWTLVFDGASNSLGHGIGAVITSPAGFHIPFTARLCFDCTNNTAEYEACIFGLEAAIDMKIKFLEVYGDSAIATCE
Tnana-235  LSDYLAHQPVDDYQPMKFEFPDEDIMHCETLVLEEGPEPGARWTLVFDGASNSLGHGIGAVITSPAGFHIPFTARLCFDCTNNTAEYEACIFGLEAAIDMKIKFLEVYGDSAIVTCE
Tnana-341  LSDYLAHQPVDDYQPMKFEFPDEDIMNCETPVLEEGPEPGARWTLVFDGASNSLGHGIGAVITSPAGFHIPFTA?LCFDCMNNTAEYEACIFGLEAAIDLKIKFLEVYGDSAIVTCE
Tnana-123  LSDYLAHQPVDDYQPMKFEFPDEDIMHCETPVLEEGPEPGARWTLVFDGASNSLGHGIGAVITSPAGFHIPFTARLCFDCTNNTTEYEACIFGLEAAIDLKIKFLEVYGDSAMVTCE
Tnana-125  LSDYLAHQPVDDYQPMKFEFPDEDIMNCETPVVEEGPEPGARCTLVFDGASNSLGHGIGAAITSPAGFHIPFTARLCFDCTNNTAEYEACIFGLEAAIDLRIKFL*VYGDSALVTCE
```
