# Supplementary material for: Obtaining retrotransposon sequences, analysis of their genomic distribution and use of retrotransposon-derived genetic markers in lentil (Lens culinaris Medik.)
Source: PLoS One. 2017 Apr 27;12(4):e0176728. doi: 10.1371/journal.pone.0176728 (PMC5407846; doi:10.1371/journal.pone.0176728)
Supplement: S1 Table — (PDF) [file pone.0176728.s005.pdf]

S1 Table

Name of primer	Sequence of Forward primer	Sequence of Reverse primer	Annealing temp. F/R (^{o}C)	Reference
Copia	5´ CARATGGAYGTNAARAC 3´	5´ CATRTCRTCNACRTA 3´	45.0	Hirochika y Hirochika, 1993
Gypsy	5´ MRNATGTGYGTNGAYTAYMG 3´	5´ RCAYTTNSWNARYTTNGCR 3´	50.0	Friedsen *et al*., 2001
Tnana 1	5´ GAAAAATMGAKCTGARTTATTCTC 3´	5' GTTCACAKGTTRCYAKTGCC 3'	52.0	This work
Tnana 2	5´ GTACACRATCTYTCWCCTGG 3´			
Tnana 3	5´ CAAAARRGYTATCGTTGCTA 3´			
PBS-1	5' ACTTGGATGCTGATACCA 3'		52.0	Kalendar *et al*., 2010
PBS-2	5' AACCGACCTCTGATACCA 3'		51.0	
PBS-3	5' GACCTAGCTCTAATACCA 3'		51.0	
PBS-4	5' GAACTTGCTCCGATGCCA 3'		51.0	
PBS-5	5' GAACCCTTGCCGATACCA 3'		51.0	
PBS-6	5' CCCCTCCTTCTAGCGCCA 3'		51.0	
PBS-7	5' CGACCTGTTCTGATACCA 3'		52.5	
PBS-8	5' TCTAGGCGTCTGATACCA 3'		52.9	
PBS-9	5' TCATGGCTCATGATACCA 3'		53.2	
PBS-10	5' TCGAGGCTCTAGATACCA 3'		51.0	
PBS-11	5' TCCCCAGCGGAGTCGCCA 3'		52.8	
PBS-12	5' AAACTGGCAACGGCGCCA 3'		52.0	
RNasah1	5' MGNACNAARCAYATHGA 3'	Adapter according to the restriction enzyme used	45.0	
RNasah2	5' GCNGAYATNYTNACNAA 3'			
LTR-Ps	5' TGAAGGAGAATTGCGACCCAAAGCGC 3'	5' TGTTGGTGTAAGCCCCTAGAGGCC 3'	50.0	Smýkal et al., 2009
LTR1	5' ATCATGCCCCTTCGTAAGGATCAC 3'	Adapter according to the restriction enzyme used	58.8	This work
LTR2	5' ATTTGGATGGGTGACCTTCTGGGA 3'		60.2	
LTR3	5' GGCGCAATGCACTTTCTAGGTGTT 3'		60.3	
LTR4	5' GTGTGACACCCTTGAATTGCATGT 3'		58.2	
SSR66R	5' GCATCACTGCAACAGACC 3'	LTR1/LTR2/LTR3/LTR4(this work, see above)	54.1	Hamwieh *et al*., 2005
SSR-AC	5' $(AC)_{10}$ 3'			This work
SSR-GT	5' $(GT)_{10}$ 3'			

Degenerate site nomenclature: N = A, C, G or T; K = G or T; M = A or C; R = A or G; S = G or C; W = A or T; Y = C or T
