# Supplementary material for: Obtaining retrotransposon sequences, analysis of their genomic distribution and use of retrotransposon-derived genetic markers in lentil (Lens culinaris Medik.)
Source: PLoS One. 2017 Apr 27;12(4):e0176728. doi: 10.1371/journal.pone.0176728 (PMC5407846; doi:10.1371/journal.pone.0176728)
Supplement: S2 Table — (PDF) [file pone.0176728.s006.pdf]

S2 Table

Model RTcount = $a*e^{b*Contig\text{-}length}$				Residuals adjusting to a Normal distribution
a	b	inter RT distance	Contigs	D statistic of Kolmogorov-Smirnov test
0.122	$1.6*10^{-5}$	>10 bp	all	D = 0.9106, $p < 2.2*10^{-16}$
0.122	$1.6*10^{-5}$	>1,000 bp	all	D = 0.9106, $p < 2.2*10^{-16}$
0.091	$1.6*10^{-5}$	>10,000 bp	all	D = 0.9106, $p < 2.2*10^{-16}$
0.074	$1.5*10^{-5}$	>50,000 bp	all	D = 0.9106, $p < 2.2*10^{-16}$
0.407	$1.2*10^{-5}$	>1,000 bp	length > 1,000 bp	D = 0.6637, $p < 2.2*10^{-16}$
0.030	$0.9*10^{-5}$	>1,000 bp	length > 10,000 bp	D = 0.7043, $p < 2.2*10^{-16}$
